# Nets versus spraying: A spatial modelling approach reveals indoor residual spraying targets *Anopheles* mosquito habitats better than mosquito nets in Tanzania

**DOI:** 10.1371/journal.pone.0205270

**Published:** 2018-10-24

**Authors:** Emily Sohanna Acheson, Jeremy Thomas Kerr

**Affiliations:** 1 Department of Geography, University of British Columbia, Vancouver, British Columbia, Canada; 2 Department of Biology, University of Ottawa, Ottawa, Ontario, Canada; Centers for Disease Control and Prevention, UNITED STATES

## Abstract

The global implementation of malaria interventions has averted hundreds of millions of clinical malaria cases in the last decade. This study assesses predicted *Anopheles* mosquito distributions across the United Republic of Tanzania before large-scale insecticide-treated net (ITN) rollouts and indoor residual spraying (IRS) initiatives to determine whether mosquito net usage by children under the age of five and IRS are targeted to areas where historical evidence indicates mosquitoes thrive. Demographic and Health Surveys data from 2011–2012 and 2015–2016 include detailed measurements of mosquito net and IRS use across Tanzania. Anopheline data are far less intensively collected, but we constructed a Maxent-built baseline mosquito habitat suitability (MHS) map (AUC = 0.872) with Tanzanian *Anopheles* occurrence records from 1999–2003. This MHS model was tested against independently-observed georeferenced *Plasmodium falciparum* cases from the Malaria Atlas Project, with ~87% of cases from 1999–2003 (n = 107) and ~84% of cases from 1985–2012 (n = 919) occurring in areas of high predicted suitability for mosquitoes. We compared the validated MHS with subsequent malaria interventions using mixed effects logistic regression. Specifically, we assessed whether *Anopheles* habitat suitability related to the frequency that ≥1 child in a household reportedly slept under a mosquito net when that intervention later became widely available, and whether IRS was reportedly applied to dwellings over a one-year period. There was no evidence that mosquito net use the night before the survey related to MHS from 2011–2012 and marginally significant evidence (p<0.05) from 2015–2016 (β = 1.466, 95% C.I. = 0.848–2.103, marginal R^2^ = 0.020, respectively). However, the likelihood of IRS treatments rose relatively strongly in the 12 months prior to both surveys (β = 13.466, 95% C.I. = 10.488–16.456, marginal R^2^ = 0.144, and β = 6.817, 95% C.I. = 5.439–8.303, marginal R^2^ = 0.136, respectively). IRS treatments have therefore been targeted more effectively than mosquito nets toward areas where anopheline habitat suitability was previously found to be high.

## Introduction

Malaria intervention methods are suggested to have averted an estimated 663 million clinical cases of malaria between 2000 and 2015, with the most widespread control method, insecticide-treated mosquito nets (ITNs), claiming 68% of the contribution [1]. Yet, the global malaria burden remains high, with 212 million estimated malaria cases worldwide and 429,000 estimated deaths in 2015 [2]. The fight against malaria is complicated by climate change, urbanization, land use changes, and behavioural changes [3]. The same factors also affect the ecology and distribution of *Anopheles* mosquitoes, the exclusive vector of malaria parasites [4], which are the core focus of malaria prevention initiatives. In comparison with the relatively widespread distribution of mosquito nets, indoor residual spraying (IRS) is the second major vector-control tool against malaria and is used largely in targeted areas only, where insecticide is applied inside dwellings on surfaces that may serve as resting places for mosquitoes, such as walls [5]. To identify gaps in malaria intervention distributions over a given area, vector-control coverage is often estimated by evaluating whether households own and use ITNs, have had IRS, or both [2]. Incomplete accessibility of malaria interventions in areas where malaria transmissions risks are high are likely to add to the malaria disease burden in affected communities. While the relationship between mosquito net ownership and high resolution *Anopheles* mosquito habitat suitability has been assessed using Geographic Information Systems (GIS), remote sensing and ecological niche modelling [6], mosquito net use by children under the age of five, as well as IRS applications, have not yet been explored using similar techniques. In addition, the spatial analysis relating reported use of interventions with a high resolution metric of anopheline mosquito habitat suitability has not previously been attempted.

Within the United Republic of Tanzania (hereafter Tanzania), an extensive ITN national plan is in place, supported by the Global Fund to Fight AIDS, Tuberculosis and Malaria (GFATM) as well as the USA President’s Malaria Initiative (PMI) [7]. As of 2004, pregnant women were provided subsidized ITNs during antenatal visits by the Tanzania National Voucher Scheme [8]. In June 2005, the U.S.-led PMI selected Tanzania for intervention programs [9, 10], leading to the development of strategies to expand ITN and IRS coverage. From 2009–2010, a mass campaign distributed ~8.7 million ITNs across Tanzania, free to families with children under five years of age [11]. In 2011, a Universal Coverage Campaign distributed another ~17.6 million ITNs nationwide, with goals to increase ITN use in the general population to 80% [7]. The GFATM set another national target to increase the proportion of Tanzanian households with at least one ITN to 90% by 2013.

The IRS campaign in Tanzania was funded by the PMI, starting in 2006 for Zanzibar and 2007 for the mainland [12]. From June 2006 through June 2011, IRS applications ran in Zanzibar on both islands of Unguja and Pemba [13]. On mainland Tanzania, IRS began in the Kagera region in 2007. By 2009, IRS applications expanded throughout all districts of the Kagera region, followed by expansions into Mwanza and Mara in 2010. Within these three areas of the Lake region, urban districts were largely excluded [13]. The goal of the National Malaria Control Programme (NMCP) Medium-Term Strategic Plan envisioned IRS being used with other vector control interventions (mainly long-lasting insecticidal nets, or LLINs), with an initial, rapid ‘knock down’ phase of targeted spraying in large geographic areas, followed by a ‘keep down/targeted’ phase of targeted spraying in large geographic areas, and then a scaled-down final ‘targeted/focal’ phase to contain focal transmission in identified hot spots [14]. Malaria risk was assessed for targeting based on a variety of malaria-related indicators, including outpatient department cases and admissions due to malaria and anemia [14]. Spraying continued from 2012 through 2015 on both Zanzibar and the mainland, but efforts were scaled down beginning in 2012 to only target malaria epidemic-prone areas [10, 12].

The nearly twenty-fold increase in malaria control financing between 2000 and 2015 [15] enabled the massive scale-up of malaria interventions over the last decade, though the distribution of the main interventions (ITNs, IRS, and prompt treatment of malaria cases with artemisinin-based combination therapy) has been uneven [1]. A predictive model of *Anopheles* mosquito habitat suitability in Tanzania, built to compare relative mosquito habitat suitability to mosquito net ownership [6], revealed that the spatial distribution of mosquito net ownership across Tanzania during the 2011–2012 AIS survey was statistically unrelated to relative mosquito habitat suitability. For this model, relative mosquito habitat suitability was calculated for Tanzania prior to 2004 when large-scale ITN rollouts were initiated [8]. The map was validated with georeferenced malaria cases from 1985–2008 and compared to georeferenced mosquito net ownership, used with permission from the DHS 2011–2012 AIDS Indicator Survey data for Tanzania. The analyses further revealed that a growing proportion of Tanzanian households owned no mosquito nets in humid, low elevation areas where relative habitat suitability for anopheline mosquitoes was highest, which includes regions of Tanzania where malaria transmission is endemic. While ITN rollouts have been broadly distributed in Tanzania, reflecting the strong commitment to malaria vector control interventions seen globally, these rollouts were least comprehensive in areas where the needs were greatest.

Along with Tanzania’s national targets and goals, the World Health Assembly endorsed its own 15-year malaria framework for all countries working to control and eliminate malaria. The framework, called the World Health Organization (WHO) Global Technical Strategy, has set goals for 2020, including reducing malaria case incidence and mortality by 40% from 2015 [2]. Tanzania shows promise in reaching most of the 40% reduction milestones set for 2020. Tanzania’s Zanzibar region (including Pemba Island and Unguja Island) are already on track to reaching the 2020 milestone in both case incidence and mortality, but the mainland of Tanzania is only on track to reach the decreased mortality goal, with less than a 10% decrease in incidence between 2010 and 2015 [2]. Though large-scale ITN rollouts appeared to have resulted in uneven ownership that declines in areas with higher malaria risk [6], have IRS applications shown similar trends? How do mosquito net use by children under the age of five and IRS coverages compare to relative *Anopheles* mosquito habitat suitability, and what improvements can be made to help reach WHO and PMI strategy goals as they approach a declining and uncertain funding horizon?

We use the validated metrics of relative habitat suitability for *Anopheles* mosquitoes in Tanzania in 2001 ([6]; before large-scale ITN rollouts, i.e. that could have been available over the period when the rollouts were being planned) to determine whether mosquito net usage by children under the age of five and IRS are targeted to areas where mosquitoes are most likely to thrive. The current study serves as a template for the use of relative *Anopheles* habitat suitability to plan and then implement control interventions. It supplements analyses of ITN and IRS coverages by adding a GIS and remote sensing perspective while also analyzing coverages in two time periods.

## Materials and methods

### Study area

**Fig 1:**
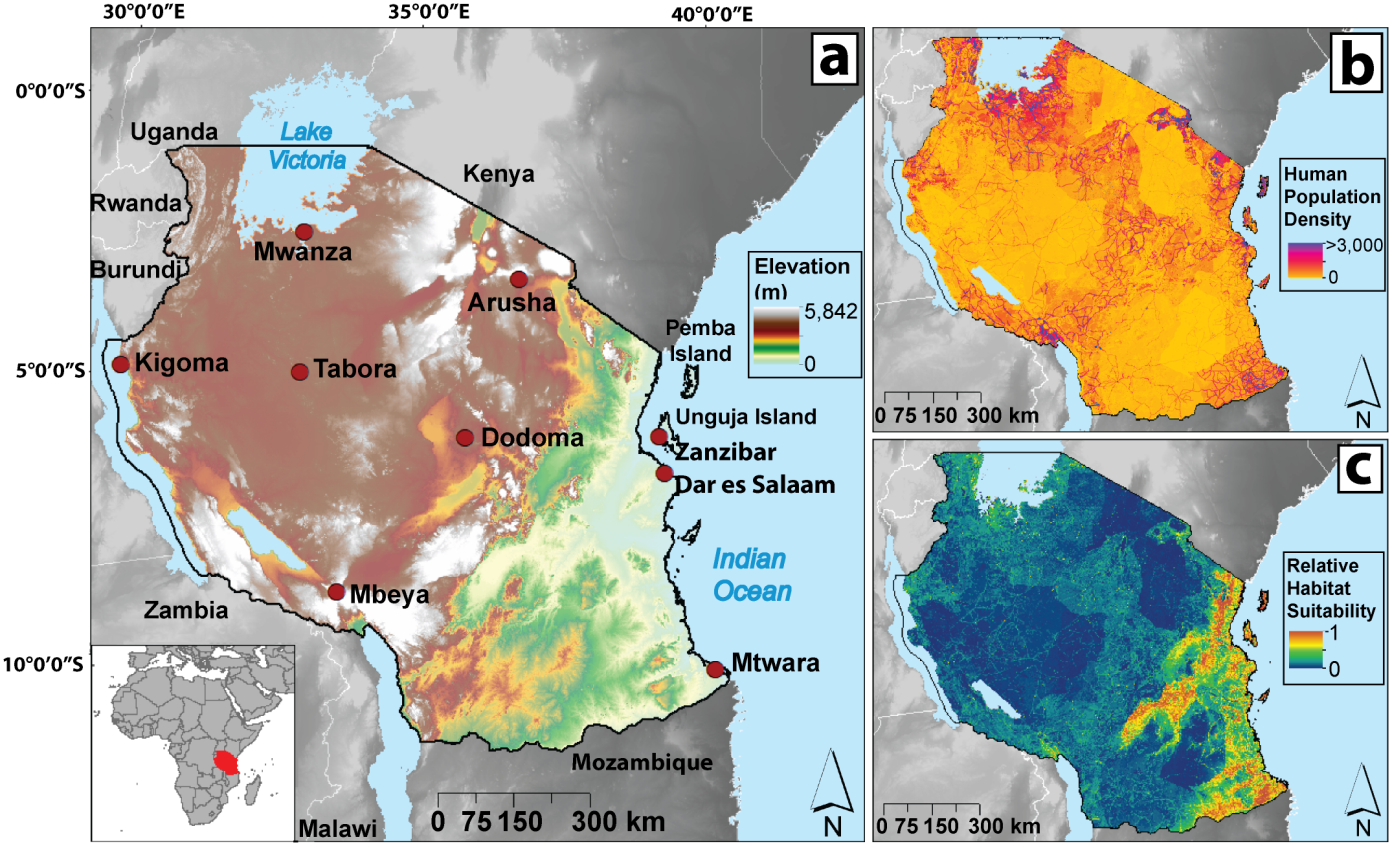
Map of the United Republic of Tanzania, human population density map, and *Anopheles* habitat suitability map. The figure shows (A) an elevation map with major cities of Tanzania and neighbouring countries. The inset map details Tanzania’s location in eastern Africa. (B) Map of human population density from LandScan (http://web.ornl.gov/sci/landscan/) with ~1 km^2^ (30 arc second) resolution. (C) The Maxent output map of relative *Anopheles* mosquito habitat suitability. The map ranges from blue to red representing low to high habitat suitability, respectively.

Tanzania and its islands in the Indian Ocean (Fig 1A), located on the east coast of Africa and occupying 886,100 km^2^ [16], are included in the study area. Its population is increasing (Fig 1B), reaching approximately 45 million in 2012 [17] and 48.8 million in 2015 [16]. The country remains one of the lowest income countries in the world, with a 2015 GDP per capita of approximately $879 U.S., equivalent to 8.7% of the world’s average [18]. The country has a tropical climate with wet and dry seasons that vary with its topography. The population distribution is extremely uneven. Nearly 80% of the population lives in low densities in arid rural areas while the remaining population occupies high-density urban areas including Dar es Salaam, with some areas of the city reaching over 3,133 people per km^2^ [19].

In 2016, 100% of Tanzanians on both the mainland and Zanzibar region were at risk of malaria [9, 10]. The most dangerous malaria parasite, *Plasmodium falciparum*, is responsible for >85% of malaria cases in Tanzania, with pregnant women and children under the age of five at the highest risk [20]. Over 26% of all outpatient attendances are due to malaria infection, with an estimated 7.7 million confirmed malaria cases annually [9]. ITN distributions prioritize pregnant women and children under the age of five [7] while IRS applications mainly target malaria epidemic-prone areas [7, 12, 13].

### Entomological records and niche model

The *Anopheles* mosquito niche model, built using Maxent software [21], v.3.3.3k, and described in detail in [6], predicted relative habitat suitability of *Anopheles* mosquitoes across Tanzania (Fig 1C). The initiation of large-scale distributions of mosquito nets in 2004 [8] required that the niche model be built using *Anopheles* coordinates from 1999–2003. This time period provided a total of 56 geographically-unique coordinates for model building, which exceeds the minimum requirements (>5) for informative predictions of species distributions [22], though combinations of all records from 1935–2003 as well as 1935–2014 yielded qualitatively similar Maxent outputs. A database of over 400 published literature sources, supplemented with unpublished data collections and the online database IR Mapper [23] from 1935 to 2014 was used for georeferenced coordinates of *Anopheles* mosquitoes. Lack of geographically-unique species-level identifications for many of the georeferenced *Anopheles* records required all recorded *Anopheles* species within the 1999–2003 time period (i.e. mosquitoes recorded as *A*. *arabiensis*, *A*. *gambiae* s.s., *A*. *gambiae* s.l., and *A*. *funestus*) to be combined for model building.

Multiple models were constructed and tested for accuracy using elevation as well as land cover, human population distribution, and variations of annual temperature and normalized difference vegetation index (NDVI) for 2001 (average, minimum, and maximum). Using data provided by the Moderate Resolution Imaging Spectroradiometer (MODIS) sensor on the Terra satellite, we collected all 8-day land surface temperature composites (MOD11A2, 1000-m resolution), all monthly NDVI composites (MOD11A3, 1000-m resolution), and land cover (MCD12Q1, 500-m resolution) for 2001. Elevation data at 90-m resolution were collected from the Shuttle Radar Topography Mission [24]. Human population distribution data for 2001 were provided by the OakRidges National Laboratory Landscan database at 30 arc second (or ~1 km) resolution [25].

To assess model accuracy, occurrence records were randomly partitioned into model training (75%) and model testing (25%), with ten replicates run for each model using tenfold cross-validation and a randomized partitioning of training and testing data in each run [6]. The raster maps of habitat suitability were then averaged to determine relative habitat suitability for each grid cell. Model accuracy was then determined using both a threshold-dependent binomial omission test as well as a threshold-independent receiver operating characteristic analysis (AUC) [21]. The binomial test of omission used a fixed threshold for habitat suitability values of 0.1, a threshold value commonly used in other studies [e.g. 26]. The omission error rate was 7.14% at the 0.1 threshold, and remained below 20% up to a threshold of 0.25 (n = 56) [6]. The internal accuracy of the model was also high for the threshold-independent analysis, with an AUC of 0.872 (where AUC values approaching 1 indicate perfect discrimination between suitable and unsuitable areas for the study species, while values near 0.5 indicate a performance no better than random) [21].

Given the set of *Anopheles* presence records and the values of the selected environmental variables across Tanzania, Maxent produced a predicted distribution of *Anopheles* mosquitoes across the country by finding the distribution of maximum entropy, constrained by the expectation that the mosquitoes’ distribution matches their empirical average over the sampled locations [21]. The resulting map used for this analysis consists of 1-km^2^ pixels of relative *Anopheles* mosquito habitat probability, rather than presence-absence of the species, where each cell ranges from 0 (i.e. low relative habitat suitability) to 1 (i.e. high relative habitat suitability). The binomial test of omission was repeated, also with a set threshold of 0.1, to test the habitat suitability map against independently-collected georeferenced cases of *Plasmodium falciparum* across Tanzania from the Malaria Atlas Project (MAP) website [27]. Model predictions of anopheline mosquito habitat suitability corresponded strongly to MAP records both from 1985–2012 (omission rate = 0.1599 for 919 geographically-unique *P*. *falciparum* records) as well as to those specifically from the study period of 1999–2003 (omission rate = 0.1321 for 107 *P*. *falciparum* records). In addition to validation against malaria prevalence rates, this model has persistently low omission error rates of anopheline observations, including those from much later (i.e. 1985–2012) and uses relatively high resolution satellite remote sensing data to generate spatially detailed predictions. Based on the fixed threshold of 0.1, our model prediction of habitat suitability was qualitatively similar to the 2001 continuous surface of predicted *P*. *falciparum* parasite rates in 2–10 year olds by the MAP [27]. However, the MAP models are developed at the continental scale and may not always be able to provide sufficient detail for within-country and within-district control efforts. As a result, our relative habitat suitability map in particular, and our method of predicting baseline mosquito habitat suitability in general, could further improve targeting for anti-malaria interventions within countries and districts by providing a higher-resolution extension to the MAP outputs.

### Malaria control survey data

Here, we use countrywide Tanzanian data from the AIDS Indicator Survey (AIS) for the 2011–2012 period and the standard DHS survey for the 2015–2016 period (with permission from the Demographic and Health Surveys (DHS) program). The 2011–2012 AIS fieldwork was conducted between December 2011 and May 2012 while the 2015–2016 DHS fieldwork was conducted between August 2015 and February 2016, implemented by Tanzania’s National Bureau of Statistics [13]. Data were used specifically from the Malaria Indicator Survey (MIS) component of the 2011–2012 AIS and 2015–2016 DHS surveys. The MIS measures malaria prevention and treatment outcomes and provides data on the household coverage of malaria interventions, including mosquito net ownership (see [6]), mosquito net use, and IRS activities, amongst others. DHS procedures and questionnaires were reviewed and approved by the ICF International Institutional Review Board. Survey details are provided in associated reports [12, 13].

Survey data are presented as georeferenced cluster points across Tanzania (i.e. points representing 17–22 nearby surveyed households). To protect respondent confidentiality, the DHS used set parameters to displace each point [28]. Points categorized as urban were displaced between 0–2 km and those categorized as rural were displaced between 0–5 km, with 1% (or every 100^th^ point) displaced between 0–10 km. Shifts in distance and direction were random. However, displacement of the points was restricted to within district (‘admin2’) levels for the 2011–2012 AIS and the 2015–2016 DHS surveys. Randomized cluster displacement could therefore cross local administrative boundaries, such as ward boundaries, but not cross DHS district boundaries.

### Mosquito net use and indoor residual spraying layers

The 2011–2012 AIS survey included data on 10,040 households and the 2015–2016 DHS survey included data on 12,563 households. The GPS dataset from each survey provided by the DHS comprised of 583 and 608 georeferenced, randomly-displaced cluster points, respectively. For the 2011–2012 dataset, eleven clusters were deleted due to missing data, with 439 rural and 133 urban clusters, and a total of 9,846 households, remaining in the final layer. Of these households, 8,533 were located on the mainland and 1,313 were located in the Zanzibar region. For the 2015–2016 dataset, one cluster was deleted due to missing data, with 427 rural and 180 urban clusters, and a total of 12,544 households, remaining in the final layer. Of these households, 10,789 were located on the mainland and 1,755 were located in the Zanzibar region.

With these two survey data sets, two malaria intervention questions were analyzed: 1) Did at least one child under the age of five use a mosquito net in the house the night before the survey was conducted? and 2) Was the dwelling sprayed for mosquitoes in the 12 months prior to the survey? Mosquito nets are defined by the DHS as any mosquito net (treated or untreated), a factory-treated net that does not require further treatment (LLIN) or a net that has been soaked with insecticide within the past 12 months [12]. The question regarding mosquito net use only looked at households in the surveyed clusters that had at least one child under the age of five and analyzed children recorded in the survey as ‘de facto’ (i.e. slept in the house the night before the survey was conducted). Data regarding responses for each child in each household were taken from the Household Member Recode data set, while data regarding household responses for IRS were taken from the Household Recode data set. Binary responses were given for each variable, with “No” given a value of 0 and “Yes” given a value of 1.

Buffer zones were created in ArcGIS v.10.1 (ESRI 2012, Redlands, CA) for each survey cluster to account for the random relocation of cluster locations for confidentiality purposes. Since cluster points were randomly displaced between 0–2 km for urban locations and 0–5 km for rural locations, buffers with a radius of 2 km were created for urban clusters and buffers with a radius of 5 km were created for rural clusters. The random relocation of clusters was restricted by district boundaries (see S1 Fig for ‘admin2’ district boundaries for each survey), so buffers that crossed into an adjacent district were made more spatially precise by being clipped along the boundary. This processing resulted in 572 cluster polygons for the 2011–2012 survey and 607 cluster polygons for the 2015–2016 survey.

### Statistical analyses

We related malaria intervention variables to a baseline relative mosquito habitat suitability map for Tanzania using R statistical software [29], v.3.0.2. The two malaria intervention layers for each survey, created in ArcGIS 10.1 and consisting of buffer zones around each cluster and clipped to ‘admin2’ district boundaries, as well as the relative habitat suitability map created in Maxent, were imported into R. R was used to calculate an average relative mosquito habitat suitability value for each unique buffer zone of 2- or 5-km radius (see S1 and S2 Files for steps in ArcGIS and R used for these processes).

Mixed effects logistic models allow analysis of multilevel (i.e. hierarchical) data with binary or ordinal outcomes, where the multilevel structure may introduce correlations among observations within a surveyed cluster (e.g. among households) or within a household (e.g. among individuals) [30]. A mixed effects model is more suitable for the multilevel data provided by the DHS than a traditional fixed effects model because it allows for analysis of the fixed effect while treating surveyed groups as a random sample from a broader population of those groups throughout Tanzania [30–32]. In this analysis, the fixed effect is relative mosquito habitat suitability and the random effects are clusters of households. Both survey questions had a binary outcome (“Yes” = 1, “No” = 0). Mixed effects logistic regression analyses were therefore conducted in R using the ‘lme4’ package to test whether relative *Anopheles* habitat suitability predicted mosquito net use by children under the age of five or IRS in the last 12 months. Each model regressed the log odds of the outcome probability on the relative mosquito habitat suitability to estimate the probability that the household answered “Yes” (i.e. 1), given the random effect.

For each question, Eq (1) was used for the mixed effects logistic regression model. This model contains one random effect and one fixed effect to compare whether the log odds of at least one child under the age of five using a mosquito net in the household the night before the survey, as well as whether the log odds of a dwelling being sprayed for mosquitoes within the last 12 months, were related to relative mosquito habitat suitability:

$$ln(\frac{p_{jk}}{1-p_{jk}})\;=\;\beta x_{k}+\;\mu +\;\gamma_{k}$$ (1)

Variables are defined as the following: *p*_*jk*_ = *P*(Y_*jk*_ = 1), the probability that the *j*th household in the *k*th cluster answers “Yes” to either having at least one child under the age of five using a mosquito net the previous night or having been sprayed for mosquitoes in the last 12 months, β = the coefficient of the fixed effect, *x*_k_ = the continuous fixed effect variable (the averaged relative mosquito habitat suitability from the buffer zone around each cluster), *μ* = the intercept, γ_k_ = the random intercept representing the random effect of the *k*th cluster. The distribution of Y_*jk*_ is *Bin*(1, *p*_*jk*_), where the mixed effects logistic regression model assumes a binomial distribution of the response variable. It is assumed that γ_k_ follows a normal distribution with a mean of 0 and variance σ^2^. For mosquito net use, we also separately tested for the additional potential random effect of households nested within surveyed clusters to compare whether the log odds of a child under the age of five using a mosquito net within a household the previous night was related to relative habitat suitability.

For both equations, the fixed effect regression coefficient β is therefore the log odds ratio of a higher malaria control effort (i.e. higher usage of mosquito nets by children under the age of five, or spraying of dwellings) versus a lower usage when *x*_*k*_ increases by one unit (in the case of this analysis, this would be an increase by 1% in relative mosquito habitat suitability), controlling for the random effect(s) in the model. Results were calculated using log odds (or logits) as opposed to odds ratios or probabilities because the log odds scale is linearized, translating to a 1 unit increase in the predictor resulting in a coefficient unit increase in the outcome, regardless of the values of other predictors. Random effects were treated as nested for the analyses, as opposed to crossed, because each individual was only surveyed in one household (i.e. the individual was not surveyed in more than one household) and, likewise, each household was only surveyed in one cluster.

In addition to providing the associated p values for each model outcome, we also report the marginal R^2^ and conditional R^2^ value using the ‘MuMIn’ package in R. The marginal R^2^ value has been proposed as being more appropriate for mixed effects models than the traditional R^2^ value, because it expresses an estimated variance explained only by fixed factors while disregarding that explained by random factors [33]. The conditional R^2^ value expresses an estimated variance explained by both the fixed and random factors. While information criteria are often used as comparison tools for mixed models (e.g. Akaike Information Criterion, Bayesian information criterion), they are used for comparing between models where they select a ‘better’ or ‘best’ model from a set of candidate models [34]. Information criteria therefore provide an estimate of relative fit of alternative models, and do not measure any sort of absolute model fit [33]. Marginal and conditional R^2^, by contrast, are extremely useful for mixed models because they provide an absolute value for model fit.

For mosquito net use, responses were calculated from a total of 8,828 surveyed children under the age of five from 5,480 households in 572 clusters from the 2011–2012 surveys. Responses were calculated from a total of 10,398 children under the age of five in 6,709 households in 607 clusters from the 2015–2016 survey. For IRS use, responses from 9,730 households were used from the 2011–2012 survey and 12,469 households were used from the 2015–2016 survey.

**Fig 2:**
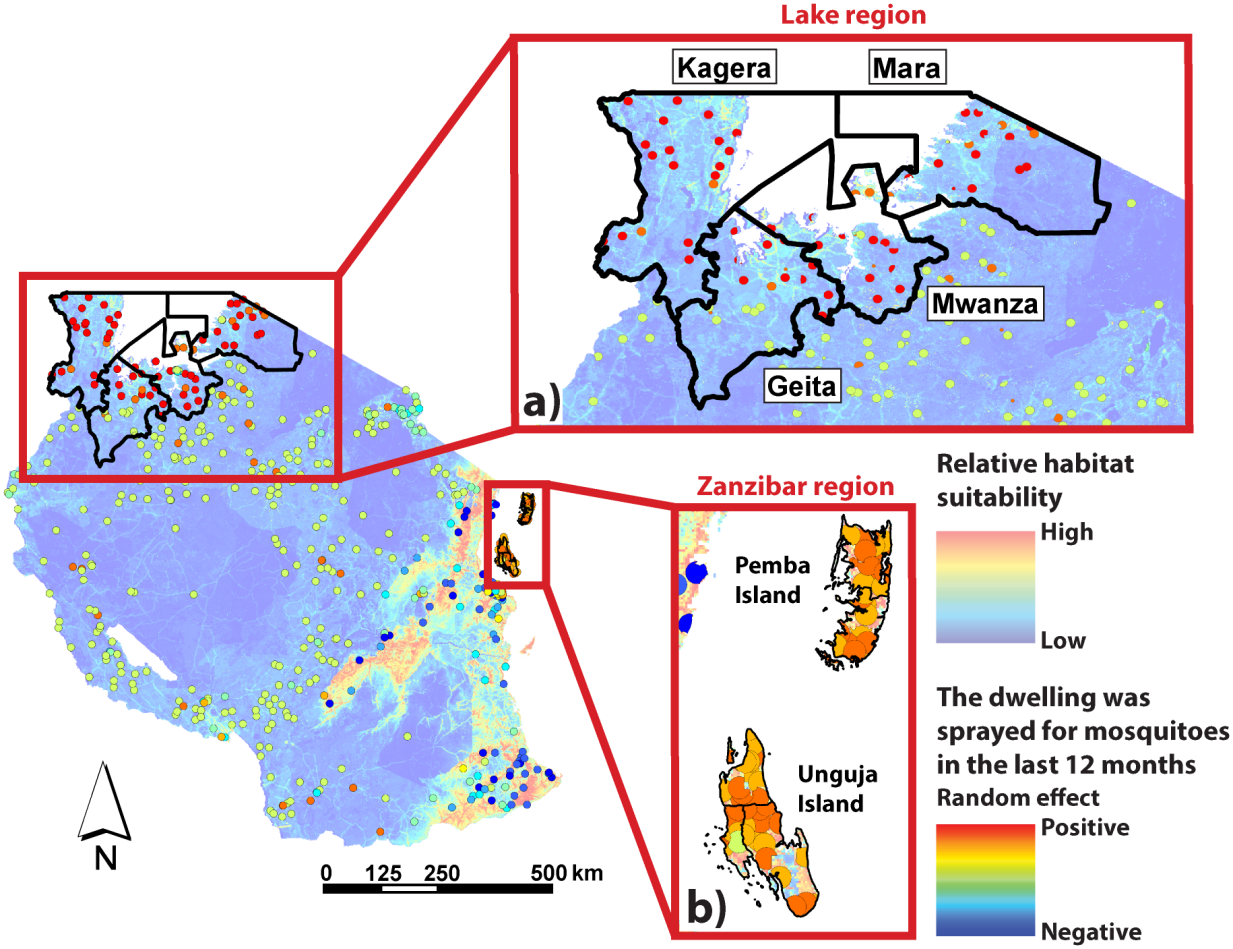
The (A) Lake and (B) Zanzibar regions, with their corresponding districts analyzed separately for dwelling spraying. The georeferenced buffer zones showing random effects contributed by each cluster are from the 2011–2012 AIS survey and are overlaid on the mosquito habitat suitability map of Tanzania. Similar regional boundaries were applied to the analysis of the 2015–2016 DHS survey data.

Due to the intensive use of IRS in the Lake and Zanzibar regions of the country compared to mosquito net distributions (Fig 2) [12, 13], analyses of dwellings being sprayed for mosquitoes in the past 12 months were conducted both countrywide and separately for a focus region of high data collection regions near Lake Victoria and on Zanzibar. According to the AIS 2011–2012 and the DHS 2015–2016 surveys [12, 13], Kagera, Mara, and Mwanza are all part of the Lake region (Fig 2A), while Pemba Island and Unguja Island are considered part of the Zanzibar region (Fig 2B), and all were specified as having been targeted by IRS initiatives. The Geita region, also part of the Lake region, was one of four new regions that formed at the time of the 2011–2012 survey design and also appears to have some IRS coverage, though the Geita region was not specified in the AIS 2011–2012 survey report as receiving IRS attention. The clipped regions of (1) Kagera, (2) Geita, (3) Mara, (4) Mwanza, (5) Pemba, and (6) Unguja were therefore analyzed separately for both surveys. Mixed effects logistic regression relating dwelling spraying and relative mosquito habitat suitability were tested for each of these six separate regions, as well as for the combined Lake region (i.e. Kagera, Geita, Mara, Mwanza), the combined Zanzibar region (i.e. Pemba Island and Unguja Island combined), and the combined Lake and Zanzibar regions, for each survey.

Since analyses of mosquito net use were not similarly restricted to specific regions, an additional analysis was conducted to test for any potential effects of undersampled districts on the countrywide models. The lowest ~10% of sampled districts were removed (i.e. all districts each containing only one sampled cluster of households) for each survey and the models were rerun. For the 2011–2012 survey comprising 148 districts, 14 districts and their associated data were deleted. For the 2015–2016 survey comprising 169 districts, 15 districts and their associated data were deleted.

To test model performance in controlling for spatial autocorrelation in residuals, we calculated Moran’s I for the residuals from each model, where a Moran’s I value of -1 indicates perfect dispersion, a value of +1 indicates perfect correlation, and a value of 0 indicates a random spatial pattern. In addition, given the mixed effects logistic regression approach used for our analysis, Moran’s I was also run on the random effect of the clusters. The random effects demonstrate the variation in malaria intervention use specific to each cluster from the model intercept. A negative random effect value means that the cluster was less likely to use the malaria intervention than the overall population when mosquito habitat suitability was held constant. Likewise, a positive random effect value means that the cluster was more likely to use the malaria intervention than the overall population when mosquito habitat suitability was held constant. Due to the targeted nature of IRS initiatives countrywide towards areas of higher malaria prevalence [14], these spatial autocorrelation tests were conducted both for the countrywide as well as the district- and region-wide analyses.

## Results

### Countrywide results for all malaria intervention questions

At a national scale, analyses of the 2011–2012 AIS dataset yielded no significant results (p>0.05) for the log odds of households having at least one child under five using a mosquito net the night before the survey, in relation to relative mosquito habitat suitability (Table 1). For the 2015–2016 DHS dataset, there were statistically significant but very weak increases in the log odds of households having at least one child under five using a mosquito net the night before the survey (β = 1.466, 95% C.I. = 0.848–2.103, p < 0.001, marginal R^2^ = 0.020, conditional R^2^ = 0.452) towards areas of higher relative mosquito habitat suitability (Table 1). Separate models were run for mosquito net use to test for the additional potential random effect of households nested within each cluster. These models, comparing whether the log odds of a child under the age of five using a mosquito net the previous night was related to relative habitat suitability, yielded qualitatively similar results for each survey period. Model results after deletion of the lowest 10% of sampled districts for each survey also yielded qualitatively similar results. Analyses revealed significant and stronger increases in the log odds of dwellings being sprayed for mosquitoes within the last 12 months toward areas with higher predicted mosquito habitat suitability for both the 2011–2012 survey (β = 13.466, 95% C.I. = 10.488–16.456, p << 1x10^-6^, marginal R^2^ = 0.144, conditional R^2^ = 0.952) and the 2015–2016 survey (β = 6.817, 95% C.I. = 5.439–8.303, p << 1x10^-6^, marginal R^2^ = 0.136, conditional R^2^ = 0.804) (Table 1).

**Table 1:** Results of the countrywide mixed effects logistic regression analyses.

Year	β	95% C.I.	S.E.	z value	p value	Marginal R^2^**	Conditional R^2^**
**Did at least one child under five used a mosquito net in the household the night before the survey?**							
**2011–2012**	-0.210	-0.672–0.257	0.236	-0.886	0.375	0	0
**2015–2016**	1.466	0.848–2.103	0.316	4.645	<0.001*	0.020	0.452
**Was the dwelling sprayed for mosquitoes in the 12 months prior to the survey?**							
**2011–2012**	13.466	10.488–16.456	1.488	9.046	<<1x10^-6^*	0.144	0.952
**2015–2016**	6.817	5.439–8.303	0.725	9.401	<<1x10^-6^*	0.136	0.804

C.I. = Confidence Interval; S.E. = Standard Error.

* Statistically significant results (p<0.05);

** R^2^ values for non-significant models are reported as 0.

**Fig 3:**
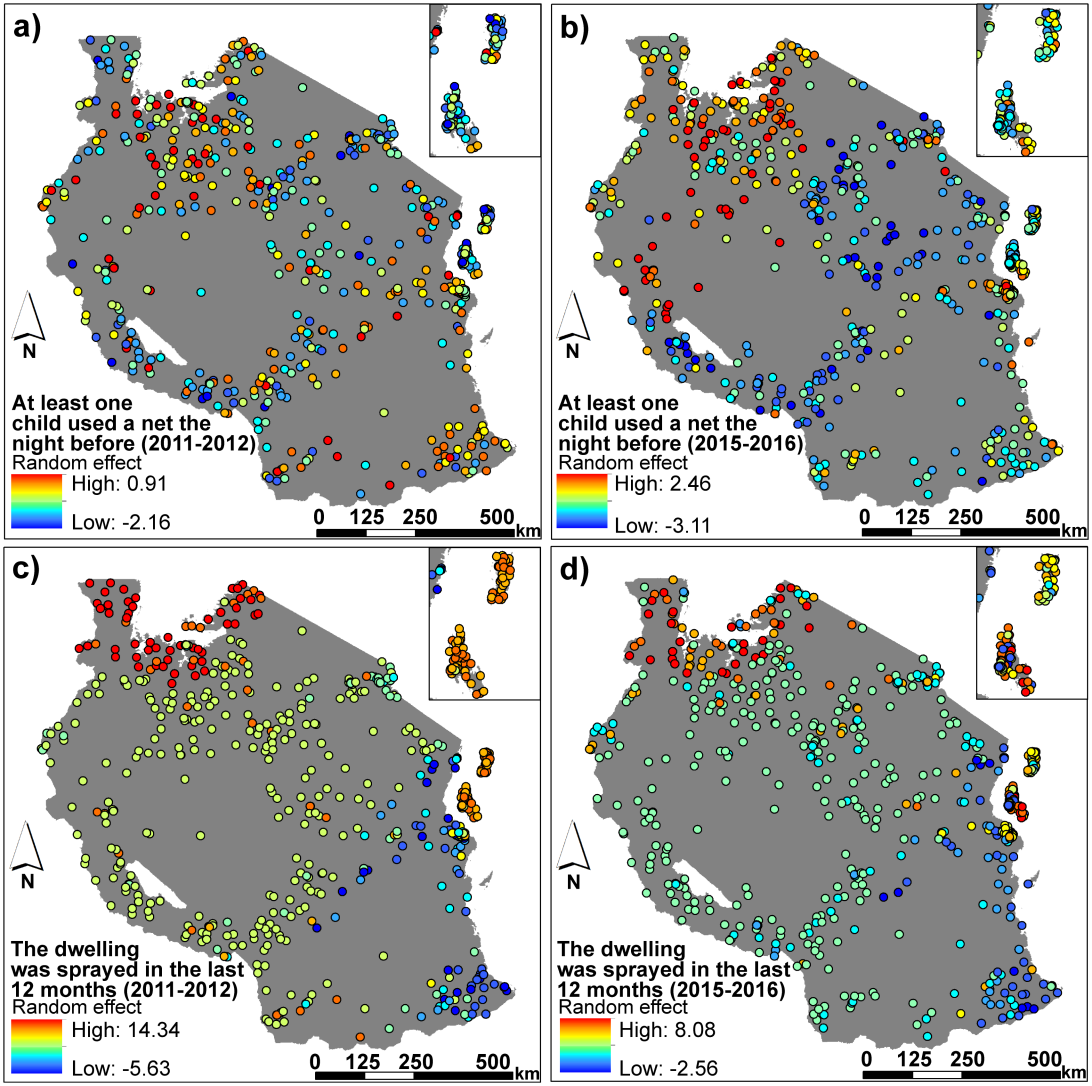
The random effect contributed by each cluster for the two malaria questions analyzed per survey. Random effects are shown for (A) and (B) Did at least one child use a mosquito net in the house the night before the survey? and (C) and (D) Was the dwelling sprayed for mosquitoes in the 12 months prior to the survey? Panels (A) and (C) show random effects from the 2011–2012 AIS survey and panels (B) and (D) show random effects from the 2015–2016 DHS survey. The random effects demonstrate the variation of each cluster from the model’s intercept (i.e. a negative random effect value means that the cluster had lower log odds of using the malaria intervention than the overall population mean when mosquito habitat suitability is held constant. The opposite applies to a positive random effect).

Spatial autocorrelation results were all statistically significant (p<0.001) but relatively low for both the model residuals and random effects of clusters across both surveys and questions (Moran’s I values ranged from 0.076–0.193, see Table 2). However, for IRS use in the 2011–2012 survey, some spatial clustering appeared to be unaccounted for by the model (Moran’s I of model residuals = 0.337, Moran’s I of random effects of clusters = 0.254) (Table 2). The random effects contributed by each cluster for each factor analyzed are shown in Fig 3.

**Table 2:** Moran’s I results for country-wide analyses. Moran’s I analyses were conducted on both model residuals as well as the random effect values of the clusters.

	Model residuals		Random effects	
Year	Moran’s I	p value	Moran’s I	p value
**Did at least one child under five used a mosquito net in the household the night before the survey?**				
**2011–2012**	0.076	<0.001	0.071	<0.001
**2015–2016**	0.168	<0.001	0.193	<0.001
**Was the dwelling sprayed for mosquitoes in the 12 months prior to the survey?**				
**2011–2012**	0.337	<0.001	0.254	<0.001
**2015–2016**	0.104	<0.001	0.149	<0.001

### Individual and combined-region results for IRS applications

Within-region models relating intervention use to models of mosquito habitat suitability yielded a surprising range of positive and negative relationships. Mixed effects logistic regression analyses demonstrated that the log odds of spraying over the previous 12 month period actually decreased toward areas with higher mosquito habitat suitability in the Mwanza region for both the 2011–2012 survey (β = -18.429, 95% C.I. = -40.051 –-1.769, p = 0.036, marginal R^2^ = 0.176, conditional R^2^ = 0.771), the 2015–2016 survey (β = -21.215, 95% C.I. = -37.382 –-9.835, p = 0.001, marginal R^2^ = 0.363, conditional R^2^ = 0.631), and on Pemba Island in the 2015–2016 survey (β = -8.512, 95% C.I. = -15.675 –-1.582, p = 0.014, marginal R^2^ = 0.046, conditional R^2^ = 0.213) (see S1 Table). However, a statistically-significant increase in the log odds of dwellings being sprayed towards areas of higher mosquito habitat suitability was seen in the Lake region in the 2011–2012 survey (β = 20.108, 95% C.I. = 4.394–32.562, p = 0.006, marginal R^2^ = 0.067, conditional R^2^ = 0.865) and in the combined Lake and Zanzibar regions in both the 2011–2012 survey (β = 9.338, 95% C.I. = 7.400–11.521, p<<1x10^-6^, marginal R^2^ = 0.327, conditional R^2^ = 0.823) and in the 2015–2016 survey (β = 3.878, 95% C.I. = 2.604–5.242, p<<1x10^-6^, marginal R^2^ = 0.115, conditional R^2^ = 0.639) (see S1 Table).

Spatial autocorrelation analyses of within-region model residuals and random effects of clusters also yielded a surprising range of positive and negative values with varying degrees of spatial autocorrelation. Moran’s I values were greater in the 2011–2012 dataset than in the 2015–2016 dataset. For the 2011–2012 survey, spatial autocorrelation analyses of model residuals were statistically significant for the Geita, Mara, and Mwanza districts and suggested some clustering (Moran’s I = 0.319, 0.336, and 0.298, respectively, p<0.001), as well as for the Lake region (Moran’s I = 0.241) and the combined Lake and Zanzibar region (Moran’s I = 0.321) (see S2 Table). Moran’s I values of the random effects of clusters yielded similar results (see S2 Table).

In the 2015–2016 survey, however, Moran’s I measurements of spatial autocorrelation analyses were often statistically significant but relatively small. Spatial autocorrelation analyses of model residuals were statistically significant for Kagera and Unguja (Moran’s I = 0.172 and 0.115, respectively, p<0.05), as well as for the Lake, Zanzibar, and combined Lake and Zanzibar region (Moran’s I = 0.054, 0.102, and 0.106, respectively, p<0.001). Moran’s I values of the random effects of clusters were also mostly relatively low and statistically significant for all districts and regions except Geita (see S2 Table).

## Discussion

Interventions are most needed—and likely to diminish malaria burdens—in areas where anopheline mosquito habitat suitability is high and consequent malaria risks increase [6]. Yet, at a national scale, anti-malaria interventions relate unevenly to mosquito habitat suitability. The likelihood of households using IRS treatments rose with MHS relatively strongly in the 12 months leading up to the 2011–2012 AIS (β = 13.466, p<<1x10^-6^, marginal R^2^ = 0.144) as well as the 2015–2016 survey (β = 6.817, p<<1x10^-6^, marginal R^2^ = 0.136), respectively. In contrast, we found no evidence that households were more likely to have at least one child under the age of five using a mosquito net the night before the survey in areas where mosquito habitat suitability was high in the 2011–2012 survey. These results are similar to those of the previous analysis of 2011–2012 mosquito net ownership [6], which found that relative mosquito habitat suitability was statistically unrelated to reported mosquito net ownership, and even decreased significantly towards areas with the highest vector habitat suitability among households with the lowest mosquito net ownership. However, analysis of the 2015–2016 survey found that households were more likely to have at least one child under the age of five using a mosquito net the night before the survey towards areas of higher relative mosquito habitat suitability (β = 1.466, p<0.001, marginal R^2^ = 0.020). The relatively stronger relationship between IRS use towards areas of increasing mosquito habitat suitability compared to mosquito net use may be due to the targeted nature of IRS initiatives, which prioritize areas and populations with high malaria risk. By targeting at-risk populations, the regions undergoing the most intensive IRS treatments are likely also areas with the highest densities and habitat suitability for anopheline mosquitoes.

Comparison between survey time periods yield similarly mixed evidence that malaria interventions have been targeted effectively. On one hand, our findings suggest that households were more likely to have at least one child under five using a net in areas of higher relative mosquito habitat suitability in the later time period. However, according to the DHS 2015–2016 Final Report [12], countrywide use of ITNs among children under the age of five peaked at 72% in the 2011–2012 survey before declining to 54% by the 2015–2016 survey. Declining ITN use might be manifest in the tendency for survey clusters to have lower odds of applying malaria interventions relative to other areas of Tanzania during the 2015–2016 surveys (Fig 3A and 3B). In addition, the marginal R^2^ value for the 2015–2016 model analysing mosquito net use by at least one child in a household was very small (marginal R^2^ = 0.020, respectively). While the model suggests households were more likely to have at least one child under five using a net as mosquito habitat suitability increased in this later time period, it also suggests this relationship is weak. By comparison, while there is a visible trend in the spatial variation of random cluster effects for IRS for the two surveys (Fig 3C and 3D), positive random cluster effects (i.e. the cluster is more likely to be applying IRS than the overall population average) appear to recede in the Lake and Zanzibar regions. These results were also confirmed by the DHS 2015–2016 Final Report [12], which reported that the percentage of countrywide households covered by IRS in the past 12 months declined from 14% in the 2011–2012 survey to only 6% in the 2015–2016 survey. However, this may be due to IRS initiatives entering the final ‘targeted’ phase of spraying to contain focal transmission in identified hot spots [14].

Ideally, mosquito net and IRS coverage would be universal, with all Tanzanians having access to at least one malaria intervention method. However, given limited resources, the next best option is to target areas with the highest mosquito habitat suitability. While countrywide mosquito net use and IRS may have decreased towards the more recent survey period in Tanzania [12], our spatial analysis offers a more encouraging outlook by suggesting that, as of 2015–2016, mosquito nets and IRS are indeed being targeted towards areas with higher relative mosquito habitat suitability. If every household had at least one child using a net or was sprayed for mosquitoes, modelling relationships between malaria intervention use and mosquito habitat suitability would be difficult (i.e. a logistic regression model would have difficulty distinguishing between answers of 0 and 1 because all answers would be 1). While this could potentially explain the non-significant trend in mosquito net use for the 2011–2012 survey period, we do not believe this was the case. Similar to mosquito net ownership in [6], mosquito net use in 2011–2012 was not universal and showed low numbers in some areas of the country (Fig 3).

In the World Malaria Report 2016, the Zanzibar region is expected to achieve the WHO Global Technical Strategy 2020 goal to reduce malaria incidence and mortality by 40% since 2015, while mainland Tanzania is only on track for the mortality goal [2]. IRS coverage is indeed less extensive on the mainland than it is in Zanzibar. As of the 2015–2016 DHS survey, IRS was more common in Zanzibar (35% of households) than on the mainland (5%), with the Lake region having the highest percentage of coverage of all mainland regions (15%) and the Kagera district having the highest coverage amongst mainland districts (25%) [12]. While our raw data also reflected these trends, the results of our district- and region-level analyses on IRS applications in relation to *Anopheles* habitat suitability were surprisingly variable. Certain regional areas were significantly (p<0.05) more likely to be sprayed for mosquitoes in the 12 months leading up to one or both surveys (the Lake region in the 2011–2012 survey, and the combined Lake and Zanzibar regions in both surveys) towards areas of higher relative mosquito habitat suitability (see S1 Table). However, the smaller district areas of Mwanza and Pemba were both significantly less likely to have been sprayed towards areas of higher mosquito habitat suitability in one or both surveys (see S1 Table). In addition, our results did not find any statistically-significant result (p>0.05) for spraying in Kagera, despite the DHS stating it had the highest coverage of all mainland districts [12].

There are several possible reasons for the variable results seen in the district- and region-level IRS analyses. First, the varying findings across districts may be due to the comparatively smaller sample sizes in these smaller areas (e.g. lowest district sample size was 19 clusters with 327 households), which may have been more susceptible to extreme values, compared to regions (e.g. lowest region sample size was 75 clusters with 1,286 households) and the entire country (the sample sizes ranged from 572 clusters with 9,730 households to 607 clusters with 12,469 households). Second, the scale down of IRS initiatives after 2012 [10, 12] may also explain the negative trends. According to the 2011–2012 AIS Final Report [13], IRS applications reached peak coverage by 2011 on the mainland and in Zanzibar. However, starting in 2012, IRS would only be applied in specific targeted areas and subsequent efforts were scaled down. Scale down was triggered in areas where ITN coverage and use appeared universal and on the basis of entomological and epidemiological evidence that scaling back IRS could be justified [35]. The declining IRS coverage may be linked with the transition from pyrethroids to more expensive insecticides, such as organophosphates, due to pyrethroid resistance in mosquitoes [2, 36, 37]. In accordance with the AIS report, the World Malaria Report 2016 [2] further reported that the global proportion of people at risk of malaria that were protected by IRS declined from its peak of 5.7% in 2010 to 3.1% in 2015, with declines seen in all WHO regions. Preliminary reports studying the scale backs of IRS coverage studied between 2008 and 2015, including a reduction of 68% of structures sprayed in Tanzania, have documented a malaria resurgence in areas of IRS withdrawal [38]. This resurgence may be due to IRS being scaled back disproportionately over geographical space, with higher mosquito habitat suitability areas experiencing more scale back than lower suitability areas within these districts and regions. This may be a third reason for the negative trends seen in some district-level analyses. Our findings also suggest that *Anopheles* habitat suitability maps may be a very useful tool to help direct IRS scale back, where scale backs could instead begin in lower *Anopheles* habitat suitability areas. A fourth reason for variability in the district- and region-level analyses may be local factors that cannot be measured using suitability models, such as awareness of mosquito net or IRS benefits among local populations, which might be a consequence of local education campaigns.

Spatial autocorrelation of model residuals and random effects of clusters was higher in the IRS coverage variable in 2011–2012 (Moran’s I of model residuals = 0.337, Moran’s I of random effects of clusters = 0.254) compared to the mosquito net use variables across both surveys (Moran’s I values ranged from 0.076–0.193, p<0.001) (Table 2), suggesting there was still spatial autocorrelation unaccounted for by the IRS models. We tested the robustness of our results to spatial autocorrelation by analyzing specific districts within the Lake and Zanzibar regions. Spatial autocorrelation of residuals and random effects of clusters remained significant but relatively small for most districts and regions, suggesting most models fit the data well. However, some clustering persisted in a few districts (see S2 Table). Multilevel models of health survey data collected from administrative boundaries or other localized areas have found higher spatial autocorrelation of model residuals compared to models that analyse health data extrapolated across a continuous surface [39]. Previous attempts have been made to create extrapolated surfaces of DHS intervention data but model fit was consistently poor when compared with observed DHS data [6]. Future analyses could supplement these point-based analyses by further attempting to create extrapolated surfaces between mosquito net and dwelling spraying and other variables to confirm the current study’s results.

The niche model was quantitatively validated, presenting with low omission error rates with *Plasmodium falciparum* malaria cases from 1985–2012 as well as qualitatively consistent predictions of *Anopheles* distributions compared to Malaria Atlas Project (MAP) predictions [27]. However, future analyses would also benefit from models built with more systematically-collected, georeferenced species-level *Anopheles* records over longer time periods and at higher spatial resolutions. Our relative mosquito habitat suitability map is informative at a countrywide scale but does not address short term or locality-specific changes in mosquito distributions or abundances that may follow mass ITN rollouts and IRS initiatives. In particular, the challenge of collecting an adequate number of geographically-unique records, as well as the lack of species-level identifications within *A*. *gambiae* s.l. for Maxent modelling led to the combination of mosquito records and species over multiple years. As a result, the map could not address the seasonality of malaria. Systematic monitoring of mosquitoes in both wet and dry seasons and over years could effectively replace the need for such a model design by separating the wet and dry seasons, but have not been undertaken. Future GIS analyses may also benefit from the addition of other factors, such as accessibility to mosquito net resources, to investigate their potential effects on mosquito net use or indoor residual spraying.

It is also important to note that the AIS and DHS fieldwork was conducted at different times of the year for the two survey periods, which would be likely to create differences in ITN use that reflected seasonal variation in mosquito activity. However, analyzing the two consecutive survey results conducted over partially overlapping time periods demonstrates relatively consistent results between mosquito net use and IRS application within each survey across countrywide and district scales. Fieldwork for the 2011–2012 AIS survey was conducted from December to May, which falls within the peak malaria season that extends from October to May (usually peaking following high rainfalls from March to May and from November to January) [40, 41]. By comparison, the fieldwork for the 2015–2016 DHS survey was initiated during the period with lowest malaria risk during the height of the dry season (August) but extended into the peak malaria period (February). Nevertheless, the likelihood of households using IRS treatments still rose significantly with increasing mosquito habitat suitability in the 12 months leading up to both surveys, and the latter survey revealed weak but significant increases in the likelihood of children using a net the night before the survey with mosquito habitat suitability while the earlier survey did not. This may therefore suggest that the potential effects of the timing of surveys does not have a significant effect at the spatial scales analyzed in this study. Future studies would benefit from comparing survey results and seasonality maps of *Anopheles* mosquitoes between the rainy and dry seasons to further add to these findings.

Models of habitat suitability will continue to play a role in assessing malaria risk and to directing malaria interventions efficiently. Such models can be improved by updating them with new environmental observations to predict how mosquito habitat suitability changes through time relative to differences in mosquito abundance. Data on *Anopheles* presences, absences, and abundances between seasons would also provide vital contributions to increasing the understanding of malaria transmission dynamics as well as enable finer-resolution analyses of the effectiveness of potentially life-saving interventions, such as mosquito net and IRS use, across regions and over time.

## Conclusions

Indoor residual spraying efforts target most at-risk areas while mosquito net efforts target most at-risk individuals. Both efforts are critical to malaria control and elimination but our spatial analysis shows that, compared to mosquito net use by children under five, IRS does target areas where anopheline mosquito abundances are expected to be higher better than mosquito nets. Previous studies have not yet been able to evaluate the degree to which mosquito net use by children under five and IRS relate to anopheline mosquito habitat suitability and consequent malaria risks. While mosquito net use can diminish malaria morbidity and mortality among the most vulnerable segments of human populations, we found mixed evidence of mosquito net use among children under five. Fortunately, new survey data suggests that likelihoods of mosquito net use among children has risen in areas where anopheline mosquito abundances are expected to be higher. In addition, IRS treatments were more strongly related to mosquito habitat suitability than mosquito net use by children under five. Additional efforts to monitor mosquito abundance would be valuable to test models and evaluate their capacity to predict changing suitability through time. Spatial modelling provides useful and informative statistical and visual tools to aid these efforts and can be applied in any area with similar disease vector occurrence records and health survey data. In the short term, however, goals to reduce malaria incidence and mortality are fast approaching. Evaluating and adapting plans to distribute interventions relative to measurable differences in mosquito habitat suitability may be valuable, not only for Tanzania but also for other malaria-endemic countries, in accelerating progress toward those goals and to reducing the malaria burden in most severely affected areas.

## Supporting information

S1 Fig Maps of ‘admin2’ districts for a) the 2011–2012 AIS survey and b) the 2015–2016 DHS survey overlaid on mosquito habitat suitability map. The ‘admin2’ districts were used to clip buffer zones to their district boundaries. For the 2011–2012 AIS survey, 148 ‘admin2’ districts were provided by the Spatial Data Repository of the DHS program (http://spatialdata.dhsprogram.com). For the 2015–2016 DHS survey, 169 ‘admin2’ districts were provided by the National Bureau of Statistics for Tanzania (http://www.nbs.go.tz/) and confirmed for use by the DHS.
(TIF)

S1 File R script and steps in ArcGIS used to create mosquito net buffer zone layers for the 2011–2012 AIS and 2015–2016 DHS surveys. Description of data: This additional file is a Word document containing the R script and the ArcGIS steps used to create the buffer zones and clip them to their district boundaries. This file can be imported directly into R for use.
(DOCX)

S2 File R script used to calculate average of habitat suitability pixels under each buffer zone. Description of data: This additional file is a Word document containing the R script used to extract the average mosquito habitat suitability values under each buffer zone. This file can be imported directly into R for use.
(DOCX)

S1 Table Mixed effects logistic regression results for individual districts and regions for IRS applications. This table was too wide for the manuscript and therefore provided as an additional file. This table contains the mixed effects logistic regression results for individual districts and regions of Tanzania relating IRS applications to predicted habitat suitability for anopheline mosquitoes. For each district or region, the first row contains results from the 2011–2012 survey and the second row contains results from the 2015–2016 survey.
(DOCX)

S2 Table Moran’s I results for districts and regions for IRS applications. This table was too wide for the manuscript and therefore provided as an additional file. This table contains the Moran’s I results for the district- and region-level analyses on indoor residual spraying. Moran’s I analyses were conducted on both the model residuals as well as on the random effect values of the clusters.
(DOCX)
