# Supplementary figures and images for: Nets versus spraying: A spatial modelling approach reveals indoor residual spraying targets Anopheles mosquito habitats better than mosquito nets in Tanzania
Source: PLoS One. 2018 Oct 24;13(10):e0205270. doi: 10.1371/journal.pone.0205270 (PMC6200228; doi:10.1371/journal.pone.0205270)

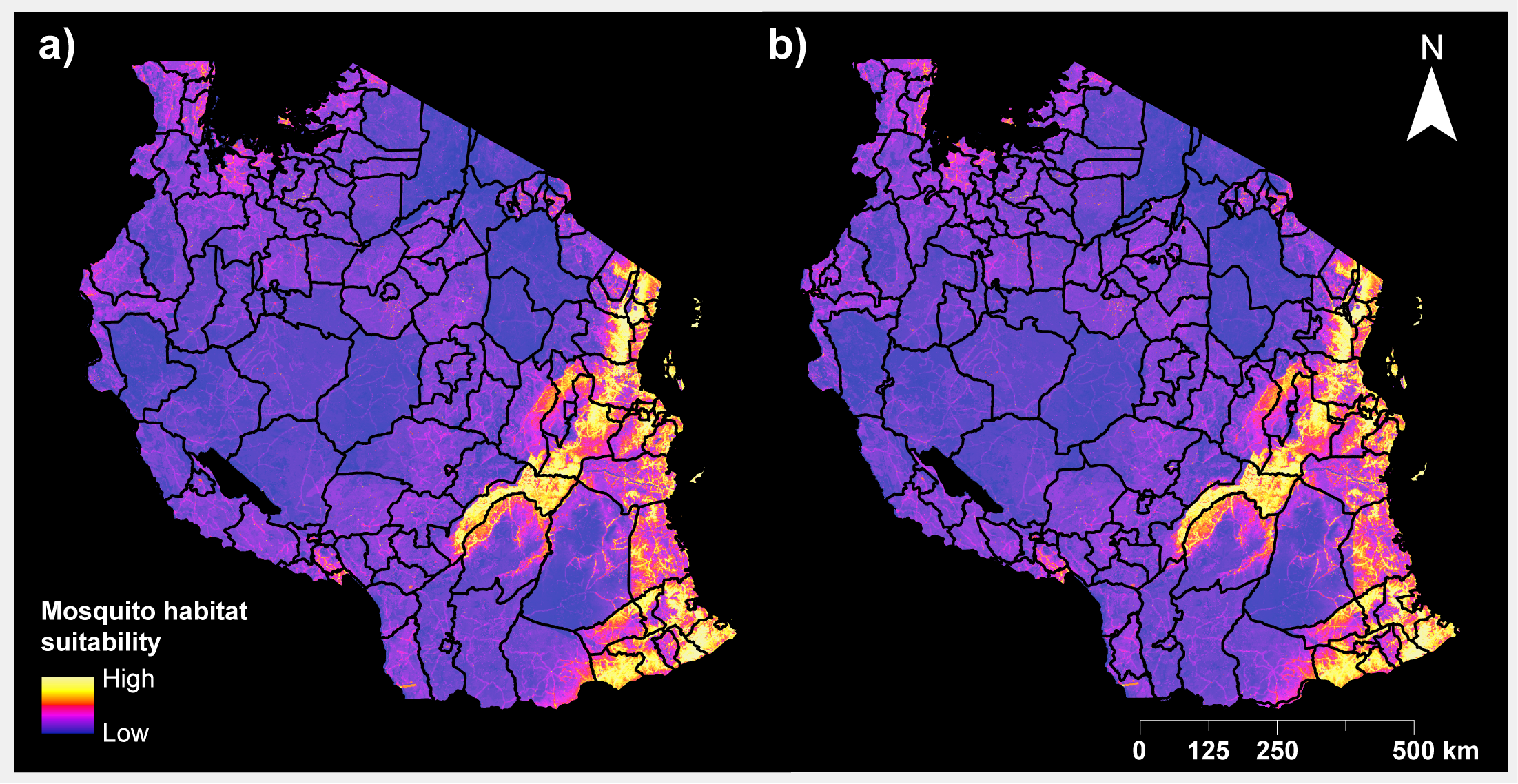

Supplement: S1 Fig — The ‘admin2’ districts were used to clip buffer zones to their district boundaries. For the 2011–2012 AIS survey, 148 ‘admin2’ districts were provided by the Spatial Data Repository of the DHS program (http://spatialdata.dhsprogram.com). For the 2015–2016 DHS survey, 169 ‘admin2’ districts were provided by the National Bureau of Statistics for Tanzania (http://www.nbs.go.tz/) and confirmed for use by the DHS. (TIF) [file pone.0205270.s001.tif]
