# Supplementary material for: Nets versus spraying: A spatial modelling approach reveals indoor residual spraying targets Anopheles mosquito habitats better than mosquito nets in Tanzania
Source: PLoS One. 2018 Oct 24;13(10):e0205270. doi: 10.1371/journal.pone.0205270 (PMC6200228; doi:10.1371/journal.pone.0205270)
Supplement: S1 File — Description of data: This additional file is a Word document containing the R script and the ArcGIS steps used to create the buffer zones and clip them to their district boundaries. This file can be imported directly into R for use. (DOCX) [file pone.0205270.s002.docx]

**S1 File: R script and steps in ArcGIS used to create mosquito net buffer zone layers for the 2011-2012 AIS and 2015-2016 DHS surveys**

#------------------------------------------------------------------------------------------------------------------#

# This document contains the annotated code used to create the layers containing the mosquito

# net use by children under the age of five per house for the 2011-2012 AIS survey data set (the same methodology can be repeated for the factors analyzing mosquito net use by at least one

# child in a household, and indoor residual spraying by households for both the 2011-2012 and

# 2015-2016 survey data sets). These scripts include how the buffer zones were clipped to district boundaries using R (<http://www.r-project.org/>). Instructions and comments start with a

# ‘#’ sign and everything written to the end of that line is read by R as a comment and not as a

# command. “Set the path” refers to specifying where the data were located in our computer.

#------------------------------------------------------------------------------------------------------------------#

**# Create the buffer zones layer in ArcGIS 10.1**

# In ArcMap 10.1, take the raw georeferenced cluster points provided by the DHS and split the

# dataset into two separate cluster shapefiles: one shapefile for urban clusters and one shapefile

# for rural clusters. For each point shapefile, use the buffer tool to create buffer zones around

# each cluster (see buffer tool here: toolboxes --> system toolboxes --> analysis tools -->

# proximity --> buffer). For rural clusters, create a buffer zone of 5 km and for urban clusters,

# create a buffer zone of 2 km. Once completed, combine the two resulting buffer shapefiles

# together using the merge tool (see merge tool here: toolboxes --> system toolboxes --> data

# management tools --> general --> merge).

**# Clip the buffer layer to district boundaries in R**

###############

# Load relevant libraries:

library(rgdal)

library(rgeos)

# Load data for the layer of 2-km- and 5-km-radius buffer zones, the layer defining the ‘admin2’ district boundaries, and the locations of the raw cluster points:

folder <- "C:\\Users\\R\\Documents"

buff <- readOGR(folder, "Combined_buffers")

reg <- readOGR(folder, "Admin2_Districts_2012")

pts <- readOGR(folder, "Clusters")

##################

# Assign each buffer its corresponding district number:

over <- over(pts, reg)

clust.reg <- cbind(pts[["DHSCLUST"]], over[["Dist_numb"]])

colnames(clust.reg) <- c("DHSCLUST", "Dist_numb")

buff[["Dist_numb"]] <- clust.reg[match(buff[["DHSCLUST"]], clust.reg[,"DHSCLUST"]), "Dist_numb"]

##################

# Manually assign district numbers to centroids in the water:

buff[buff[["DHSCLUST"]] == 732, "Dist_numb"] <- 40

buff[buff[["DHSCLUST"]] == 238, "Dist_numb"] <- 2

buff[buff[["DHSCLUST"]] == 430, "Dist_numb"] <- 93

##################

# Clip buffers according to its district:

outList <- vector("list", length = length(buff))

for(i in 1:length(buff)){

if(is.na(buff@data[i, "Dist_numb"])){

outList[[i]] <- buff[i,]@polygons[[1]]

}else{

distNo <- buff@data[i, "Dist_numb"]

distRow <- which(reg[["Dist_numb"]] == distNo)

int <- gIntersection(buff[i,], reg[distRow,])

outpoly <- int@polygons[[1]]

outpoly@ID <- buff[i,]@polygons[[1]]@ID

outList[[i]] <- outpoly}

}

newbuff <- SpatialPolygonsDataFrame(SpatialPolygons(outList), data = buff@data)

##################

# Save output:

writeOGR(newbuff, dsn=“C:\\Users\\R\\Documents”, layer="newbuff", driver = "ESRI Shapefile")
