# Supplementary material for: Nets versus spraying: A spatial modelling approach reveals indoor residual spraying targets Anopheles mosquito habitats better than mosquito nets in Tanzania
Source: PLoS One. 2018 Oct 24;13(10):e0205270. doi: 10.1371/journal.pone.0205270 (PMC6200228; doi:10.1371/journal.pone.0205270)
Supplement: S2 File — Description of data: This additional file is a Word document containing the R script used to extract the average mosquito habitat suitability values under each buffer zone. This file can be imported directly into R for use. (DOCX) [file pone.0205270.s003.docx]

**S2 File: R script used to calculate average of habitat suitability pixels under each buffer zone**

#------------------------------------------------------------------------------------------------------------------#

# This document contains the annotated code used to calculate the average pixel value under

# each buffer zone (overlapping areas act as a separate zone with their own averaged value)

# using R (http://www.r-project.org/).

# Instructions and comments start with a number sign (#) and everything written to the end of

# that line is read by R as a comment, not a command. “Set the path” refers to specifying where

# the data were located in our computer.

#------------------------------------------------------------------------------------------------------------------#

##################

# Load relevant libraries:

library(rgdal)

##################

# Set the path to import the habitat suitability layer for all mosquito species:

suit2001<-raster(“C:\\Users\\R\\Documents\\Anopheles.asc”)

##################

# Set the path to import the bed net polygon data for 2011-2012:

bednetPolygon<-readOGR(dsn=“ C:\\Users\\R\\Documents”, layer="newbuff")

plot(bednetPolygon)

##################

# Compare bed nets and suitability:

suitVals <- extract(suit2001, bednetPolygon)

suitAvg <- sapply(suitVals, mean, na.rm = TRUE)
