# Supplementary material for: Nets versus spraying: A spatial modelling approach reveals indoor residual spraying targets Anopheles mosquito habitats better than mosquito nets in Tanzania
Source: PLoS One. 2018 Oct 24;13(10):e0205270. doi: 10.1371/journal.pone.0205270 (PMC6200228; doi:10.1371/journal.pone.0205270)
Supplement: S1 Table — This table was too wide for the manuscript and therefore provided as an additional file. This table contains the mixed effects logistic regression results for individual districts and regions of Tanzania relating IRS applications to predicted habitat suitability for anopheline mosquitoes. For each district or region, the first row contains results from the 2011–2012 survey and the second row contains results from the 2015–2016 survey. (DOCX) [file pone.0205270.s004.docx]

**S1 Table.** **Mixed effects logistic regression results for district- and region-level analyses.** Results of the analyses of individual districts and regions of Tanzania relating IRS applications to predicted habitat suitability for anopheline mosquitoes are shown below. For each district or region, the first row contains results from the 2011-2012 survey and the second row contains results from the 2015-2016 survey.

| **Geographic region** | **‘Admin 1’ district** | **β** | **95% C.I.** | **S.E.** | **z value** | **p value** | **Marginal R^2^**** | **Conditional R^2^**** |
| --- | --- | --- | --- | --- | --- | --- | --- | --- |
| **District** | | | | | | | | |
| Lake | Kagera | -2.913 | -15.133 – 8.852 | 5.517 | -0.528 | 0.598 | 0 | 0 |
|  |  | 1.972 | -7.993 – 11.560 | 4.480 | 0.440 | 0.660 | 0 | 0 |
|  | Mara | -1.755 | -13.571 – 10.186 | 5.620 | -0.312 | 0.755 | 0 | 0 |
|  |  | -7.755 | -23.065 – 5.286 | 6.623 | -1.171 | 0.242 | 0 | 0 |
|  | Mwanza | -18.429 | -40.051 – -1.769 | 8.774 | -2.100 | 0.036* | 0.176 | 0.771 |
|  |  | -21.215 | -37.382 – -9.835 | 6.379 | -3.326 | 0.001* | 0.363 | 0.631 |
|  | Geita | 23.702 | -18.036 – 76.480 | 20.757 | 1.142 | 0.254 | 0 | 0 |
|  |  | -3.361 | -17.678 – 11.604 | 6.929 | -0.485 | 0.628 | 0 | 0 |
| Zanzibar | Pemba | 2.358 | -6.898 – 10.381 | 3.982 | 0.592 | 0.554 | 0 | 0 |
|  |  | -8.512 | -15.675 – -1.582 | 3.451 | -2.466 | 0.014* | 0.046 | 0.213 |
|  | Unguja | -2.081 | -6.762 – 2.575 | 2.320 | -0.897 | 0.370 | 0 | 0 |
|  |  | -4.181 | -13.010 – 4.526 | 4.281 | -0.977 | 0.329 | 0 | 0 |
| **Region** | | | | | | | | |
| Lake |  | 20.108 | 4.394 – 32.562 | 7.331 | 2.743 | 0.006* | 0.067 | 0.865 |
|  |  | 3.019 | -4.290 – 10.552 | 3.716 | 0.812 | 0.417 | 0 | 0 |
| Zanzibar |  | 1.762 | -1.696 – 5.302 | 1.752 | 1.006 | 0.315 | 0 | 0 |
|  |  | -3.725 | -8.824 – 1.404 | 2.527 | -1.474 | 0.141 | 0 | 0 |
| Lake and Zanzibar |  | 9.338 | 7.400 – 11.521 | 1.035 | 9.025 | <<1x10^-6* | 0.327 | 0.823 |
|  |  | 3.878 | 2.604 – 5.242 | 0.667 | 5.813 | <<1x10^-6* | 0.115 | 0.639 |

C.I. = Confidence Interval; S.E. = Standard Error. * Statistically significant result (p<0.05); ** R^2^ values for non-significant models are reported as 0.
