# Supplementary material for: Nets versus spraying: A spatial modelling approach reveals indoor residual spraying targets Anopheles mosquito habitats better than mosquito nets in Tanzania
Source: PLoS One. 2018 Oct 24;13(10):e0205270. doi: 10.1371/journal.pone.0205270 (PMC6200228; doi:10.1371/journal.pone.0205270)
Supplement: S2 Table — This table was too wide for the manuscript and therefore provided as an additional file. This table contains the Moran’s I results for the district- and region-level analyses on indoor residual spraying. Moran’s I analyses were conducted on both the model residuals as well as on the random effect values of the clusters. (DOCX) [file pone.0205270.s005.docx]

**S2 Table. Moran’s I results for the district- and region-level analyses on indoor residual spraying.** Moran’s I analyses were conducted on both the model residuals as well as on the random effect values of the clusters.

| **Model residuals** | | | | | **Random effects** | | | |
| --- | --- | --- | --- | --- | --- | --- | --- | --- |
|  | **2011-2012** | | **2015-2016** | | **2011-2012** | | **2015-2016** | |
|  | **Moran’s I** | **p value** | **Moran’s I** | **p value** | **Moran’s I** | **p value** | **Moran’s I** | **p value** |
| **District** | | | | | | | | |
| Geita | 0.319 | <<1x10^-6^* | 0.050 | 0.161 | 0.246 | <0.001* | 0.048 | 0.181 |
| Kagera | -0.115 | 0.259 | 0.172 | 0.003* | -0.076 | 0.742 | 0.235 | <0.001* |
| Mara | 0.336 | <0.001* | -0.009 | 0.580 | 0.241 | <0.001* | 0.130 | 0.017* |
| Mwanza | 0.298 | <<1x10^-6^* | -0.0003 | 0.424 | 0.354 | <<1x10^-6^* | 0.096 | 0.036* |
| Pemba | -0.019 | 0.780 | 0.049 | 0.086 | -0.008 | 0.653 | 0.089 | 0.014* |
| Unguja | -0.028 | 0.872 | 0.115 | <0.001* | 0.031 | 0.150 | 0.137 | <0.001* |
| **Region** | | | | | | | | |
| Lake | 0.241 | <0.001* | 0.054 | 0.001* | 0.226 | <0.001* | 0.106 | <<1x10^-6^* |
| Zanzibar | 0.023 | 0.197 | 0.102 | <0.001* | 0.035 | 0.139 | 0.119 | <0.001* |
| Lake and Zanzibar | 0.321 | <0.001* | 0.106 | <<1x10^-6^* | 0.201 | <0.001* | 0.114 | <<1x10^-6^* |

* Statistically significant result (p<0.05)
